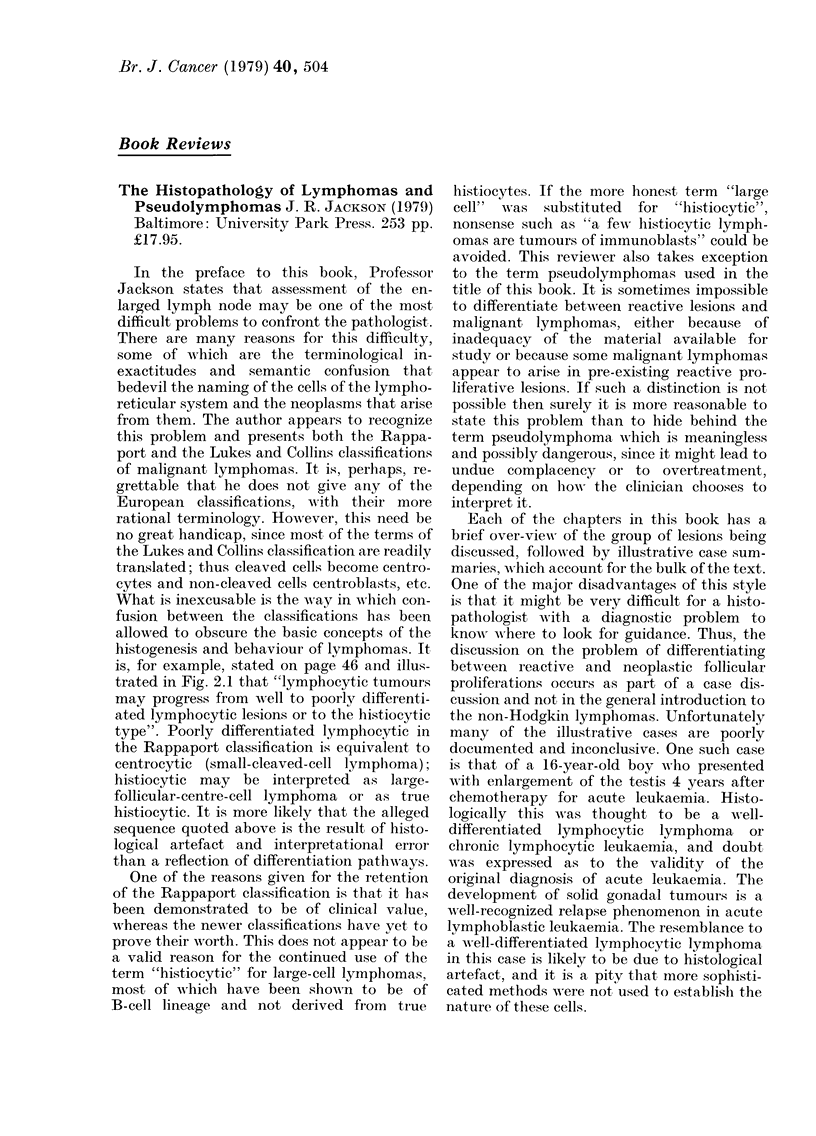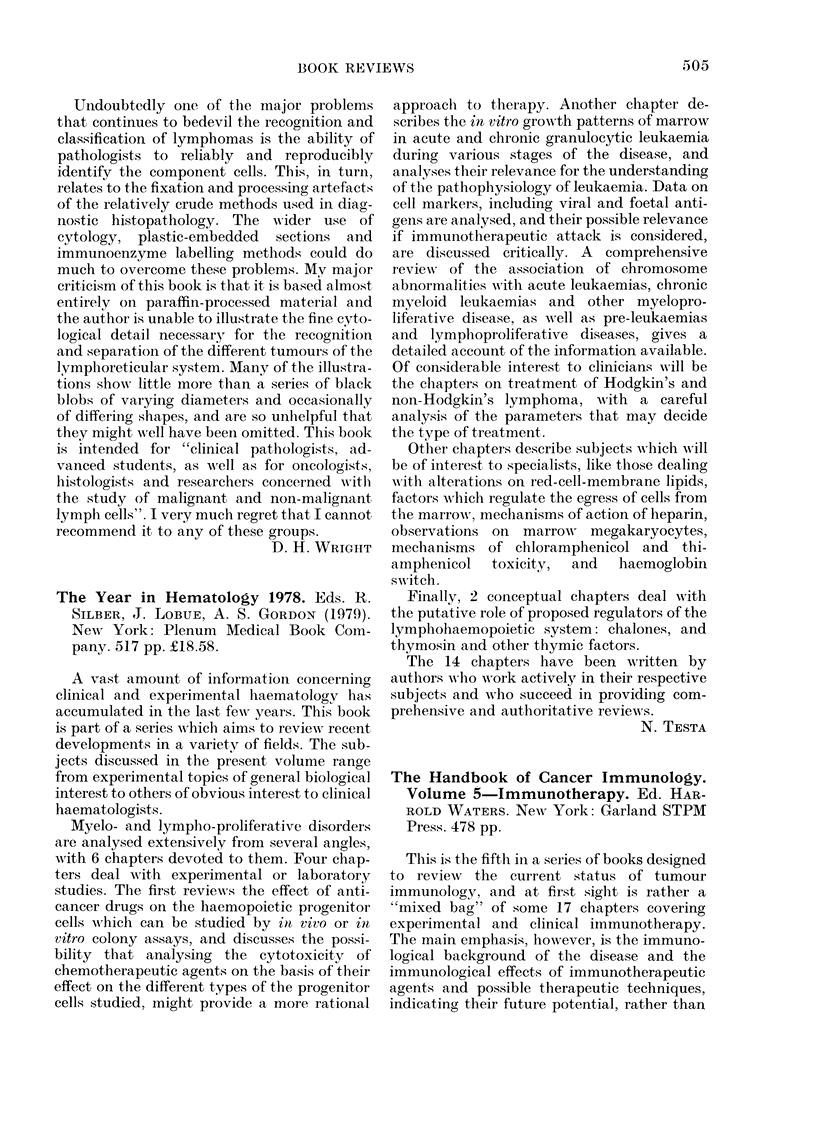# The Histopathology of Lymphomas and Pseudolymphomas

**Published:** 1979-09

**Authors:** D. H. Wright


*Br. J. Cancer* (1979) **40**, 504

### *Book Reviews*

**The Histopathology of Lymphomas and Pseudolymphomas** J. R. Jackson (1979) Baltimore: University Park Press. 253 pp. £17.95.

In the preface to this book, Professor Jackson states that assessment of the enlarged lymph node may be one of the most difficult problems to confront the pathologist. There are many reasons for this difficulty, some of which are the terminological inexactitudes and semantic confusion that bedevil the naming of the cells of the lymphoreticular system and the neoplasms that arise from them. The author appears to recognize this problem and presents both the Rappaport and the Lukes and Collins classifications of malignant lymphomas. It is, perhaps, regrettable that he does not give any of the European classifications, with their more rational terminology. However, this need be no great handicap, since most of the terms of the Lukes and Collins classification are readily translated; thus cleaved cells become centrocytes and non-cleaved cells centroblasts, etc. What is inexcusable is the way in which confusion between the classifications has been allowed to obscure the basic concepts of the histogenesis and behaviour of lymphomas. It is, for example, stated on page 46 and illustrated in Fig. 2.1 that "lymphocytic tumours may progress from well to poorly differentiated lymphocytic lesions or to the histiocytic type". Poorly differentiated lymphocytic in the Rappaport classification is equivalent to centrocytic (small-cleaved-cell lymphoma); histiocytic may be interpreted as large-follicular-centre-cell lymphoma or as true histiocytic. It is more likely that the alleged sequence quoted above is the result of histological artefact and interpretational error than a reflection of differentiation pathways.

One of the reasons given for the retention of the Rappaport classification is that it has been demonstrated to be of clinical value, whereas the newer classifications have yet to prove their worth. This does not appear to be a valid reason for the continued use of the term "histiocytic" for large-cell lymphomas, most of which have been shown to be of B-cell lineage and not derived from true histiocytes. If the more honest term "large cell" was substituted for "histiocytic", nonsense such as "a few histiocytic lymphomas are tumours of immunoblasts" could be avoided. This reviewer also takes exception to the term pseudolymphomas used in the title of this book. It is sometimes impossible to differentiate between reactive lesions and malignant lymphomas, either because of inadequacy of the material available for study or because some malignant lymphomas appear to arise in pre-existing reactive proliferative lesions. If such a distinction is not possible then surely it is more reasonable to state this problem than to hide behind the term pseudolymphoma which is meaningless and possibly dangerous, since it might lead to undue complacency or to overtreatment, depending on how the clinician chooses to interpret it.

Each of the chapters in this book has a brief over-view of the group of lesions being discussed, followed by illustrative case summaries, which account for the bulk of the text. One of the major disadvantages of this style is that it might be very difficult for a histopathologist with a diagnostic problem to know where to look for guidance. Thus, the discussion on the problem of differentiating between reactive and neoplastic follicular proliferations occurs as part of a case discussion and not in the general introduction to the non-Hodgkin lymphomas. Unfortunately many of the illustrative cases are poorly documented and inconclusive. One such case is that of a 16-year-old boy who presented with enlargement of the testis 4 years after chemotherapy for acute leukaemia. Histologically this was thought to be a well-differentiated lymphocytic lymphoma or chronic lymphocytic leukaemia, and doubt was expressed as to the validity of the original diagnosis of acute leukaemia. The development of solid gonadal tumours is a well-recognized relapse phenomenon in acute lymphoblastic leukaemia. The resemblance to a well-differentiated lymphocytic lymphoma in this case is likely to be due to histological artefact, and it is a pity that more sophisticated methods were not used to establish the nature of these cells.

Undoubtedly one of the major problems that continues to bedevil the recognition and classification of lymphomas is the ability of pathologists to reliably and reproducibly identify the component cells. This, in turn, relates to the fixation and processing artefacts of the relatively crude methods used in diagnostic histopathology. The wider use of cytology, plastic-embedded sections and immunoenzyme labelling methods could do much to overcome these problems. My major criticism of this book is that it is based almost entirely on paraffin-processed material and the author is unable to illustrate the fine cytological detail necessary for the recognition and separation of the different tumours of the lymphoreticular system. Many of the illustrations show little more than a series of black blobs of varying diameters and occasionally of differing shapes, and are so unhelpful that they might well have been omitted. This book is intended for "clinical pathologists, advanced students, as well as for oncologists, histologists and researchers concerned with the study of malignant and non-malignant lymph cells". I very much regret that I cannot recommend it to any of these groups.

D. H. WRIGHT